# Supplementary material for: Molecular Basis of Inhibitory Mechanism of Naltrexone and Its Metabolites through Structural and Energetic Analyses
Source: Molecules. 2022 Aug 2;27(15):4919. doi: 10.3390/molecules27154919 (PMC9369988; doi:10.3390/molecules27154919)
Supplement: Supplementary file 1 [file molecules-27-04919-s001.zip › molecules-1808433-supplementary.pdf]

SUPPLEMENTARY MATERIAL

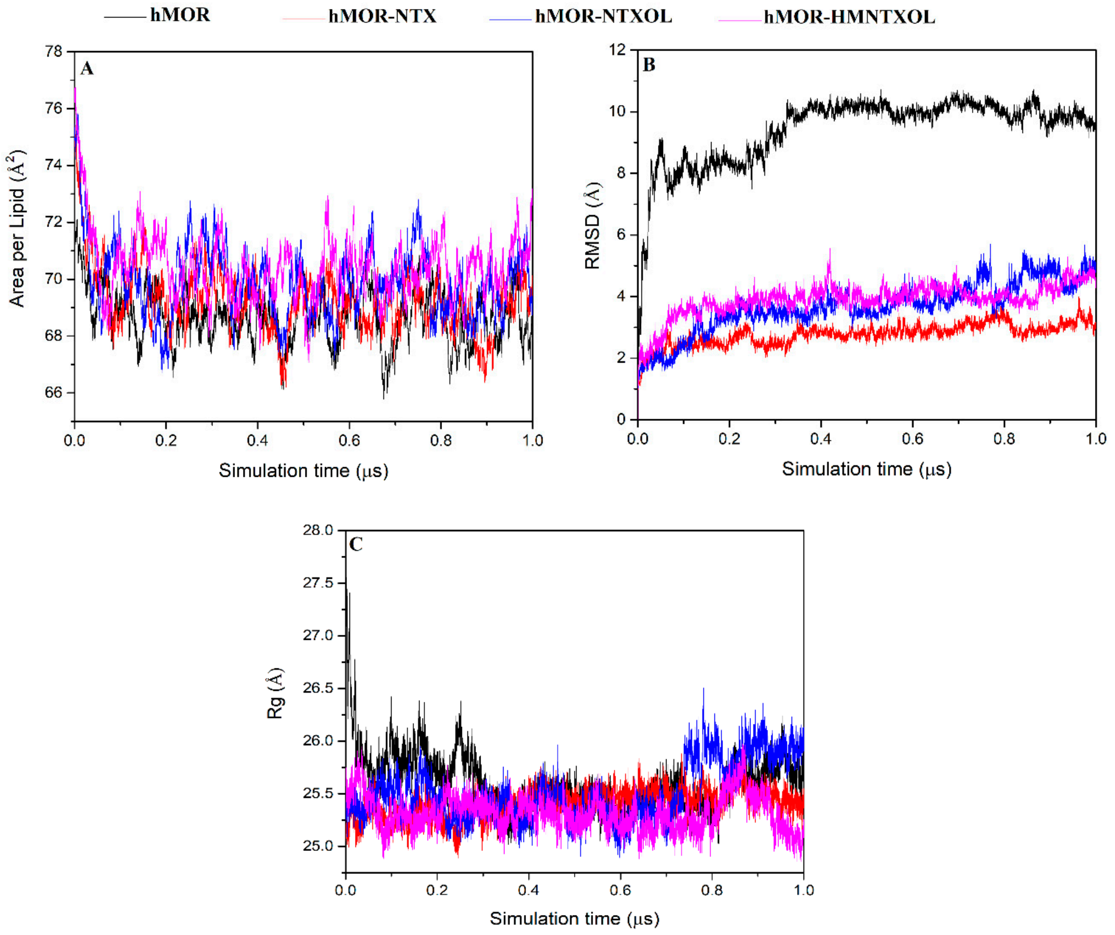

Figure S1. Area per lipid, root means squared deviation (RMSD), and radius of gyration (Rg) analysis of hMOR-ligand systems. Area per lipid (A), RMSD (B), and Rg (C) of hMOR-naltrexone, hMOR-NTXOL, and hMOR-HMNTXOL.

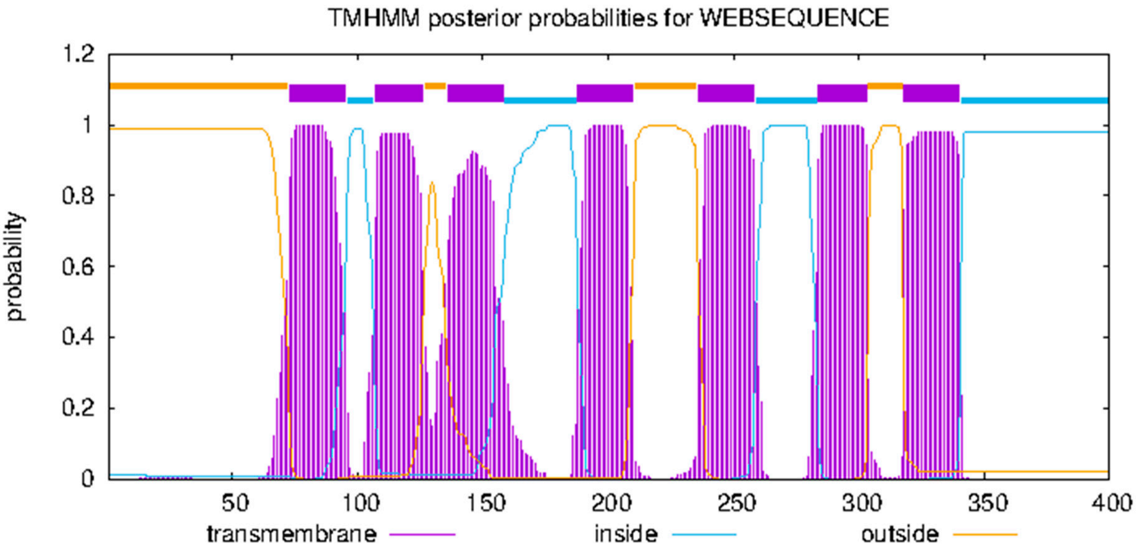

Figure S2. Prediction of the transmembrane helices in hMOR using TMHMM 2.0 server [44].

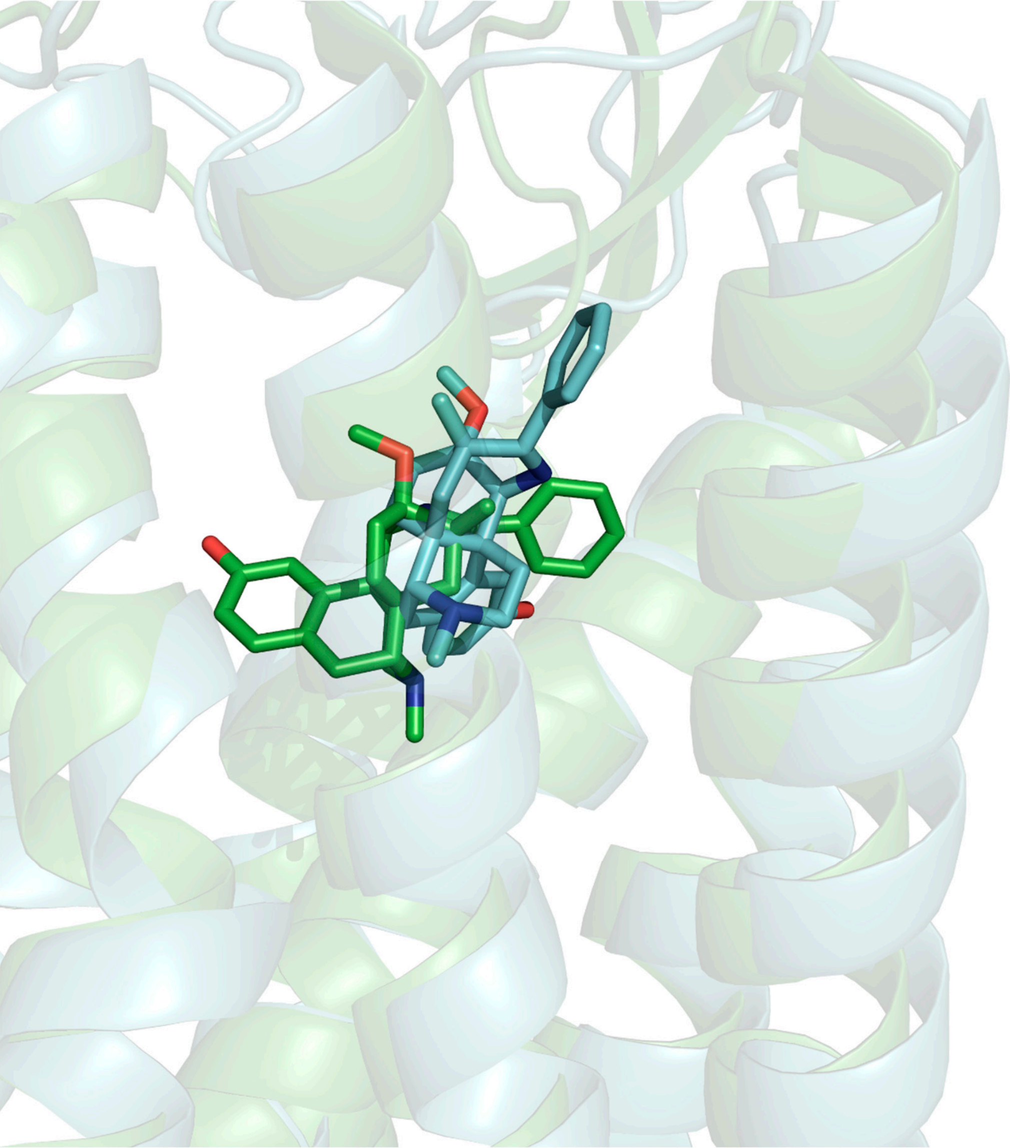

Figure S3. Molecular docking of the co-crystallized ligand (green) in mMOR (PDB entry 5C1M_chainA) in the most populated hMOR conformer (cyan) obtained through MD simulations.

Table S1. Per-residue free energy for hMOR coupled to naltrexone, NTXOL and HMNTXOL (values kcal/mol).

Residue	hMOR-NTX	hMOR-NTXOL	hMOR-HMNTXOL
L114		-0.131	
A115	-0.383	-1.173	-0.496
L118		-0.280	
A119	-0.771	-1.128	-0.812
T120		-0.119	-0.158
T122	-0.189	-0.114	-0.117
L123	-0.147		-0.138
Q126	-0.300		-0.181
N129	-0.196		
I148	-0.184		
Y150			-0.805
N152	-0.206	-0.533	
M153	-1.780	-1.551	-3.050
F154		-0.152	-0.134
T155		-0.240	
S156		-0.417	
I157		-1.016	-0.229
T159		-0.142	
L160		-0.462	
V238			-0.730
A242		-0.247	-0.129
V290		-0.158	
C294	-0.351	-0.210	

W295	-1.572	-1.176	-0.757
I298	-1.184	-0.731	-1.725
H299	-0.218		-0.556
V302			-0.345
C323			-0.374
I324	-1.216	-0.271	-1.382
A325	-0.168		
L326	-0.297	-0.143	-0.218
G327	-1.547	-0.319	-0.445
Y328	-2.774	-0.991	-1.522
T329	-0.219	-0.167	
N330	-0.876	-1.566	-0.160
S331	-0.316	-0.650	-0.439
L333		-0.156	
N334		-1.942	-0.228
